# Personality Traits and Burnout in Emergency Medicine Residents

**DOI:** 10.5811/westjem.21139

**Published:** 2025-01-30

**Authors:** Brendan Freeman, Lukasz Cygan, Laura Melville, Theodore Gaeta

**Affiliations:** * Staten Island University Hospital | Northwell Health, Department of Emergency Medicine, Staten Island, New York; † New York-Presbyterian Brooklyn Methodist Hospital, Department of Emergency Medicine, Brooklyn, New York

## Abstract

**Background:**

Burnout is prevalent in medical training, and some data indicates certain personality types are more susceptible. The criterion reference for measurement of burnout is the Maslach Burnout Inventory (MBI), which scores three factors: emotional exhaustion (EE); depersonalization (DP); and personal accomplishment (PA). Emotional exhaustion most closely correlates with burnout. Studies have yet to evaluate a link between burnout markers and certain personality traits in emergency medicine (EM) residents. The personality traits of openness, agreeableness, extraversion, conscientiousness, and neuroticism can be measured with a 50-item International Personality Item Pool (IPIP) Big 5 survey. Our goal in this study was to be the first to examine the relationship between personality traits and burnout among EM residents and guide future research on potential predictors of burnout and targeted interventions for resident well-being.

**Methods:**

This was an observational, cross-sectional study conducted in March and April of 2023 in an urban, Level II trauma center, involving all EM residents at a three-year residency program. Two surveys, the IPIP and MBI-Human Services Survey, were distributed to all residents, and their responses were anonymous. We calculated raw/mean scores and standard deviations for each personality trait/burnout measure and compared them by the Pearson correlation coefficient.

**Results:**

All 38 residents completed the surveys. A total of 31% of the cohort reported high exhaustion, 13% reported high DP, and 42% reported low PA. Two of 38 (5%) residents reported the combination of high EE, high DP, and low PA. There was a statistically significant negative correlation between conscientiousness and EE (*n* = 38; Pearson *r* = −0.40, *P* < 0.001) and a positive correlation between conscientiousness and PA (*n* = 38; Pearson *r* = 0.36, *P* = 0.03).

**Conclusion:**

In our sample, residents who were more conscientious reported experiencing lower levels of emotional exhaustion and a greater sense of personal accomplishment. Programs may cautiously explore the potential of assessing resident personality traits as part of broader efforts to identify predictors of burnout, but further research with larger, multicenter, longitudinal samples is needed to corroborate these results. The small sample size and single-center design may limit generalizability of these findings, and the use of self-reported measures introduces the risk of response bias.

Population Health Research Capsule What do we already know about this issue?

*In non-emergency medicine settings, high neuroticism, low agreeableness, low conscientiousness, low openness, and low extraversion are associated with burnout.*

What was the research question?

*In emergency medicine residents, how do the “Big Five” personality traits correlate with burnout markers?*

What was the major finding of the study?

*Conscientiousness is negatively correlated with emotional exhaustion (Pearson *r* = −0.40, *P* < 0.001, while positively correlated with personal achievement (r = 0.36, *P* = 0.03, N = 38).*

How does this improve population health?

*Identifying burnout-predictive traits could help target interventions and support resident well-being and better patient care.*

## INTRODUCTION

Burnout has emerged as a focal point for many residency programs, given its pervasiveness and severity throughout all phases of medical training.^1^ ^–^ ^3^ Due to prolonged exposure to elevated stress levels, burnout manifests through symptoms such as irritability, fatigue, cynicism, and detachment. This phenomenon holds particular relevance within the realm of emergency medicine (EM), a field known to report elevated levels of burnout. The etiology of this problem within EM is multifaceted and related to factors such as working environment (eg, physical layout and conditions, administrative tasks), shift work, violence in the workplace, exposure to infectious disease, patient volume, clinical variability, staffing, and the life-and-death decision-making inherent to the specialty.

The practice of EM hinges significantly upon interpersonal interactions, adding an additional layer of complexity to the phenomenon of burnout in the emergency physician. Because of the nature of EM, personality traits may play a more significant role in predicting burnout than in other settings. Existing investigations into this association are small-scale studies conducted with non-emergency physicians and have identified correlations of various measures of personality traits with burnout markers.^4^ ^–^ ^9^ In general, high neuroticism, low agreeableness, low conscientiousness, low openness, and low extraversion are associated with burnout.^10^ While certain personality traits may confer resilience or susceptibility to the challenges inherent in EM, the precise nature of this relationship remains underexplored within the EM literature.

The gold standard for burnout measurement is the Maslach Burnout Inventory (MBI), which measures emotional exhaustion (EE), depersonalization (DP), and personal accomplishment (PA).^11^ Among these, EE has emerged as being most closely correlated with the presence of burnout.^12^ ^,^ ^13^ Various adaptations of this inventory have been devised and validated, of which, the Human Services Survey (HSS) is the most applicable to healthcare workers.

Personality assessment within academic studies often relies on the framework of the “Big Five” traits, delineated by Goldberg (1992).^14^ These traits encompass openness, agreeableness, extraversion, conscientiousness, and neuroticism. Openness can be understood on a scale of inventive/curious to consistent/cautious. Conscientiousness ranges from efficient/organized to extravagant/careless. Agreeableness ranges from friendly/compassionate to critical/rational. Extraversion is defined as outgoing/energetic vs solitary/reserved. Lastly, neuroticism ranges from sensitive/nervous to resilient/confident. Measurements of these traits have been adapted and validated for numerous research studies.^15^ ^–^ ^19^ One form of this is the 50-item International Personality Item Pool (IPIP) representation of the Goldberg markers for the Big-Five factor structure.^20^

While these personality traits exhibit relative stability and maturation by the age of 30, their potential protective or predictive roles in mitigating burnout among resident physicians remain underexplored, particularly within the context of EM.^21^ The scarcity of studies directly investigating this relationship in emergency physicians underscores the imperative for dedicated research initiatives aimed at elucidating the interplay between personality traits and burnout within the high-stress environment characteristic of EM. Consequently, our goal was to be the first to examine the relationship between personality traits and burnout among EM residents and guide future research on potential predictors of burnout and targeted interventions for resident well-being.

## METHODS

This was an observational, cross-sectional study conducted in March and April 2023 that involved all EM residents in a three-year residency program at an urban, Level II trauma center. All residents were offered inclusion in the study via a single survey emailed to their work emails. A total of five emails were sent during the study months for recruitment. No other recruitment methods were used. Subjects were consented and completed two sequential online surveys administered in a single session: the 50-item IPIP representation of the Goldberg markers for the Big-Five factor structure and the MBI-HSS. The principal investigator (BF) selected the order of the surveys and administered the personality assessment first to avoid any potential priming effects from the burnout inventory. Additionally, the IPIP is more time intensive than the MBI and, thus, may require more attention. Answers were secured and anonymous.

We calculated raw/mean scores and standard deviations for each personality trait/burnout measure and compared them by Pearson correlation coefficient. Results were analyzed by BF using Microsoft Excel (Microsoft Corporation. Redmond, WA) and Python (Python Software Foundation, Wilmington, DE). This study received institutional review board approval.

## RESULTS

All 38 residents completed both surveys. The mean, SD, correlation coefficients and confidence intervals are reported in the Table. Thirty-one percent of the cohort reported high exhaustion, 13% reported high depersonalization, and 42% reported a low sense of personal accomplishment. Two of 38 (5%) residents reported the combination of high EE, high DP, and low PA. While there were no statistically significant differences in EE, DP, or PA across postgraduate year (PGY) levels, PGY-1 residents had higher overall mean scores of EE compared to PGY-2 residents and higher mean DP scores compared to both PGY-2 and PGY-3 residents. All PGY levels consistently reported high levels of PA.

**Table.** Correlation coefficient matrix with confidence intervals comparing the burnout factors of emotional exhaustion, depersonalization, and personal accomplishment with five key personality traits.

Variable	Openness	Conscientiousness	Extraversion	Agreeableness	Neuroticism
EE	−0.16	−0.46*	0.04	−0.02	0.08
	[−0.41, 0.21]	[−0.68, −0.17]	[−0.28, 0.35]	[−0.33, 0.3]	[−0.24, 0.39]
DP	−0.15	−0.27	−0.13	−0.11	0.19
	[−0.43, 0.19]	[−0.54, 0.04]	[−0.43, 0.19]	[−0.41, 0.22]	[−0.14, 0.47]
PA	0.09	0.36*	0.07	−0.09	−0.02
	[−0.23, 0.39]	[0.05, 0.60]	[−0.25, 0.38]	[−0.40, 0.23]	[−0.34, 0.29]

Values in square brackets indicate the 95% confidence interval for each correlation. * = *P* < 0.05.

*EE*, emotional exhaustion; *DP*, depersonalization; *PA*, personal accomplishment.

**Figure 1.**
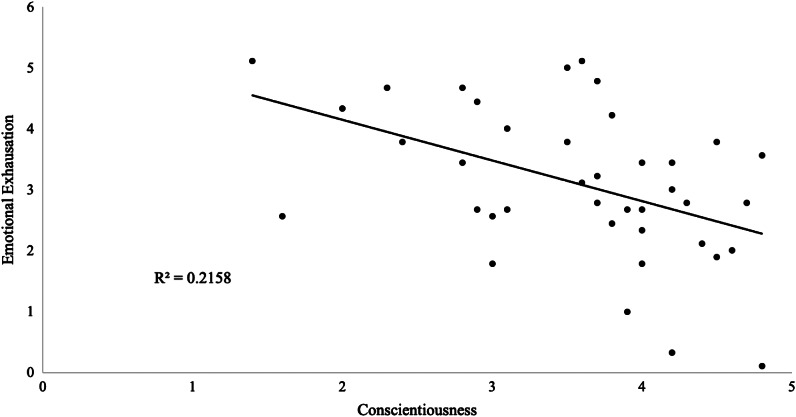
Negative correlation between conscientiousness and emotional exhaustion in emergency medicine residents (*n* = 38; Pearson’s *r* = −0.40, *P* < 0.001).

**Figure 2.**
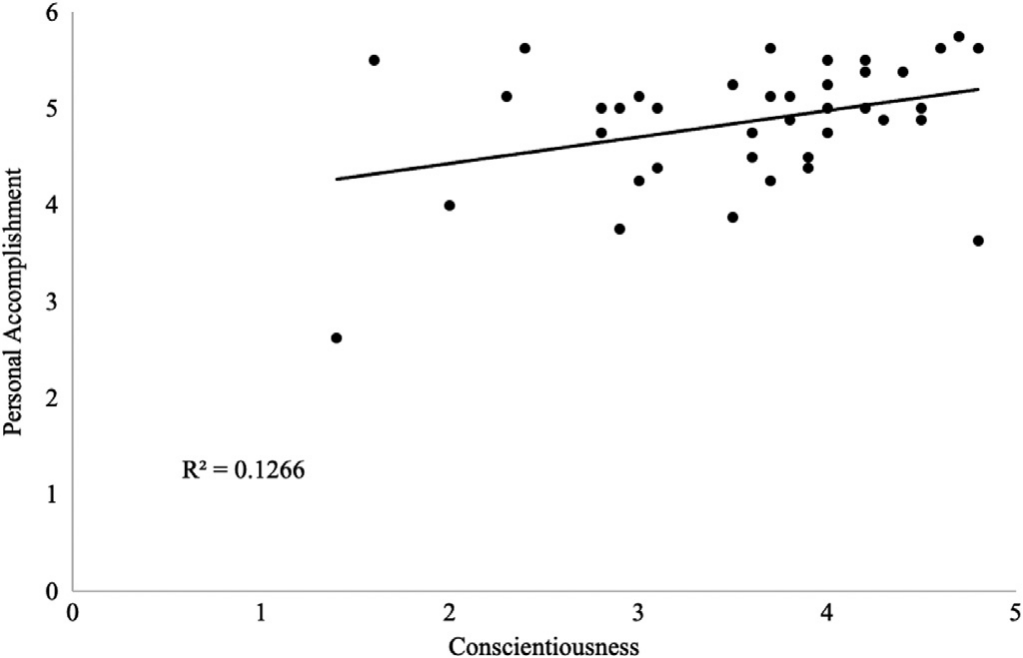
Positive correlation between conscientiousness and sense of personal accomplishment in emergency medicine residents (*n* = 38; Pearson’s *r* = 0.36, *P* = 0.03).

There was a statistically significant negative correlation between conscientiousness and EE (Figure 1, *n* = 38; Pearson’s *r* = −0.40, *P* < 0.001), which persisted across all PGY levels. Additionally, a near-significant positive correlation was observed between conscientiousness and PA (Figure 2, *n* = 38; Pearson’s *r* = 0.36, *P* 0.03), which also persisted across all PGY levels. No other statistically significant correlations were found between personality traits and burnout measures, regardless of PGY level. For all correlations, we considered Bonferroni adjustment (alpha < 0.003).

## DISCUSSION

The negative correlation between conscientiousness and EE suggests that certain personality characteristics may serve as protective factors against burnout in EM residents. This finding is consistent with previous research conducted in various occupational settings.^21^ Conscientious individuals tend to be diligent, organized, and achievement oriented, traits that may buffer against the emotional toll of demanding work environments. In the context of EM where residents are frequently exposed to high-stress situations and long hours, the ability to maintain order and efficiency in their work may contribute to lower levels of EE. Furthermore, even though it was not below the Bonferroni adjusted alpha, there was a near-significant positive correlation between conscientiousness and sense of PA, which seems to suggest that cultivating these traits in residents may even be protective of burnout and contribute to personal and professional satisfaction andcareer longevity.

Taken together, these results offer actionable insights that residency leadership can leverage to support the well-being and resilience of EM residents. Specifically, the identification of conscientiousness as a protective factor against EE suggests that interventions aimed at fostering conscientious behaviors among residents may help mitigate the risk of burnout. Conscientiousness has been identified as a trait that can be enhanced with interventions such as mindfulness and life-skills training.^22^ ^,^ ^23^ More research is needed to determine which interventions are most effective and durable, but residency programs could consider incorporating targeted training sessions or workshops focusing on organizational skills, time-management strategies, and stress- management techniques to cultivate conscientious traits among residents. Additionally, while we used anonymous data in this study, residency leadership may consider implementing personality trait assessments as part of confidential resident evaluations or onboarding processes. Occult burnout may present with poor performance or even unprofessional behavior. Thus, personality trait assessment could be used to initiate conversations about burnout.

While personality traits may play a role in shaping individuals’ susceptibility to burnout, organizational factors, workload, support systems, and coping mechanisms also exert significant influences. Future research should aim to explore these multifaceted interactions comprehensively, incorporating longitudinal studies to track the trajectories of burnout and personality development over time as they relate to EM residents. Additionally, qualitative research methods could provide valuable insights into the subjective experiences of residents and the contextual factors that contribute to burnout in EM training programs.

While this study provides valuable insights into the relationship between personality traits and burnout among EM residents, it represents just one factor among many. Addressing burnout in this population requires a multifaceted approach that considers both individual characteristics and systemic factors within the residency education environment. By gaining a deeper understanding of these dynamics, we can develop more effective interventions to support the well-being and resilience of EM residents.

## LIMITATIONS

The small sample size and single-center design limit the generalizability of the findings to residents in other settings, such as rural or community hospitals. Selection bias is also an important consideration as residents may have chosen this specific program based on individual preferences, such as geographic location, which could introduce variability unrelated to clinical experience or program type. The timing of the study was chosen to align with a relatively lower period of stress in our residency program across all PGY levels, but the results may differ between the periods of the same academic year, calendar year, or clinical rotations. Additionally, response bias is a factor, as the measures evaluated here are self-reported and participants’ responses may not accurately reflect their true characteristics. More longitudinal data will be needed to fully understand the nature of the correlations between burnout scores and personality traits observed in this cross-sectional study.

## CONCLUSION

In our sample, residents who were more conscientious had lower levels of emotional exhaustion and a greater sense of personal accomplishment. Programs may cautiously explore the potential of assessing resident personality traits as part of broader efforts to identify predictors of burnout, but further research with larger, multicenter, longitudinal samples is needed to corroborate these results.
